# Seroepidemiological Survey of the Antibody for Severe Acute Respiratory Syndrome Coronavirus 2 with Neutralizing Activity at Hospitals: A Cross-sectional Study in Hyogo Prefecture, Japan

**DOI:** 10.31662/jmaj.2020-0094

**Published:** 2021-01-14

**Authors:** Koichi Furukawa, Jun Arii, Mitsuhiro Nishimura, Lidya Handayani Tjan, Anna Lystia Poetranto, Zhenxiao Ren, Salma Aktar, Jing Rin Huang, Silvia Sutandhio, Yukiya Kurahashi, Arisa Nishino, Shiho Shigekuni, Yuichiro Takeda, Kenichi Uto, Keiji Matsui, Itsuko Sato, Yoshiaki Inui, Kazuo Endo, Yoshiyuki Kosaka, Toshiaki Oota, Jun Saegusa, Yasuko Mori

**Affiliations:** 1 Division of Clinical Virology, Center for Infectious Disease, Kobe University Graduate School of Medicine, Kobe, Japan; 2 Department of Clinical Laboratory, Kobe University Hospital, Kobe, Japan; 3 Hyogo Prefectural Nishinomiya Hospital, Nishinomiya, Japan; 4 Hyogo Prefectural Amagasaki General Medical Center, Amagasaki, Japan; 5 Hyogo Prefectural Kobe Children’s Hospital, Kobe, Japan; 6 Hyogo Prefecture Health Promotion Association, Kobe, Japan

**Keywords:** SARS-CoV-2, COVID-19, epidemiology, seroprevalence, neutralizing activity, Japan, Hyogo

## Abstract

**Introduction:**

The coronavirus disease 2019 (COVID-19) pandemic is spreading rapidly all over the world. The Japanese government lifted the state of emergency, announced in April 2020, on May 25, but there are still sporadic clusters. Asymptomatic patients who can transmit severe acute respiratory syndrome coronavirus 2 (SARS-CoV-2) cause some of these clusters. It is thus urgent to investigate the seroprevalence of antibodies against SARS-CoV-2 and their neutralizing activity. We conducted a cross-sectional study of >10,000 samples at hospitals in Hyogo Prefecture, Japan.

**Methods:**

Between August 6 and October 1, 2020, we collected samples of residual blood from the patients who visited or were admitted to five hospitals and a foundation in Hyogo. We tested the samples for antibodies against SARS-CoV-2 by electrochemiluminescence immunoassay (ECLIA) and chemiluminescent enzyme immunoassay (CLEIA). Sera that were positive by ECLIA or CLEIA were analyzed by an immunochromatographic (IC) test and neutralizing activity assay.

**Results:**

We tested 10,377 samples from patients aged between 0 and 99 years old; 27 cases (0.26%) were positive on the ECLIA, and 51 cases (0.49%) were positive on CLEIA. In the 14 cases that tested positive on both ECLIA and CLEIA, the positive rates on the IC test and for neutralizing activity were high (85% and 92%, respectively). In 50 cases (0.48%) that were positive by either ECLIA or CLEIA, the corresponding rates were low (20% and 6%, respectively). The positive rate of neutralizing antibody was 0.15%.

**Conclusions:**

These results indicate that most Hyogo Prefecture residents still do not have antibodies and should avoid the risk of incurring a SARS-CoV-2 infection. Two or more antibody tests should be required for seroepidemiological studies of the antibody for SARS-CoV-2, and a neutralizing activity assay is also essential.

## Introduction

In December 2019, the coronavirus disease 2019 (COVID-19) pandemic caused by severe acute respiratory syndrome coronavirus 2 (SARS-CoV-2), occurred in Wuhan, China ^(1)^. Since then, there have been 37,109,851 confirmed cases of COVID-19 worldwide, including 1,070,355 deaths reported to the World Health Organization (WHO), as of October 12, 2020 ^(2)^. The first case in Japan was reported in January 2020, followed by the first outbreak on a giant cruise ship called the Diamond Princess. The number of cases in Japan has continued to increase, with 89,652 infected and 1,631 deaths as of October 13, 2020. The Japanese government announced a pandemic state of emergency on April 7, 2020. It was lifted on May 25, but sporadic clusters of COVID-19 are still being reported in multiple regions, raising concerns about the virus’s spread.

**Figure 1.**
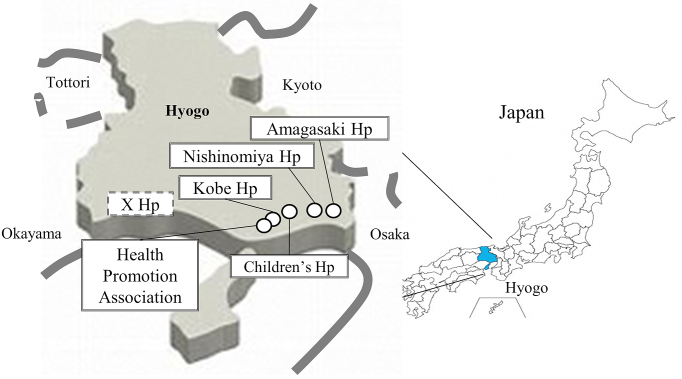
Map of Hyogo Prefecture and the six participating facilities. Hyogo Prefecture, with a population of ~5.4 million, is in the Kansai region of Japan and adjacent to Osaka, one of the severe acute respiratory syndrome coronavirus 2 (SARS-CoV-2) endemic areas in Japan.

An accurate estimation of the prevalence of COVID-19 requires antibody tests in asymptomatic individuals, and more information about the neutralizing activity of the antibodies is needed to predict epidemics in the coming winter. The accuracy of the several currently available antibody tests is unclear ^(3), (4)^. In June 2020, the Ministry of Health, Labor and Welfare of Japan randomly selected ~3,000 residents by gender and age in each of the three prefectures of Tokyo, Osaka, and Miyagi and conducted an epidemiological survey of the seroprevalence of antibodies for SARS-CoV-2. The survey revealed that 0.10%, 0.17%, and 0.03% of the residents were seropositive for SARS-CoV-2 in Tokyo, Osaka and Miyagi, respectively, suggesting that most people still do not have antibodies against SARS-CoV-2 with neutralizing activity ^(5)^. Herein, we analyzed >10,000 serum samples from the hospitals and foundation located in Hyogo Prefecture, which is in the Kansai region of Japan with a population of roughly 5.4 million people. The Prefecture of Hyogo is adjacent to Osaka, which is one of the SARS-CoV-2 endemic areas in Japan (Figure 1). We performed three different antibody tests including a neutralizing activity assay to determine the seroprevalence of antibodies for SARS-CoV-2 in Hyogo Prefecture.

## Materials and Methods

### Sample collection

From August 6 to October 1 2020, residual blood of patients who visited or were admitted to the five hospitals and a foundation in Hyogo Prefecture were selected and tested for antibodies against SARS-CoV-2. The six participating facilities were Kobe University Hospital (Ko), anonymous X Hospital (X), Hyogo Prefectural Nishinomiya Hospital (NI), Hyogo Prefectural Amagasaki General Medical Center (AM), Hyogo Prefecture Health Promotion Association (HE), and Hyogo Prefectural Kobe Children’s Hospital (CH), which are located from west to east in Hyogo Prefecture. The average number of outpatients per day at each hospital was as follows: Ko 2,000; X 1,100; NI 650; AM 1,800; HE 100 or CH 350. The respective number of samples collected at each hospital was as follows: Ko 2,048 (1,045 males); X 2,000 (989 males); NI 2,010 (904 males); AM 2,000 (1,001 males); HE 1,000 (596 males) or CH 1,319 (689 males) (5,184 males of 10,377 samples in total). The median age at each facility was 67, 69, 69, 70, 47 or 8 years old (median 62 years old).

### Antibody measurements

#### Immunochromatographic test

The Innovita 2019-nCoV Ab Test (Innovita Biological Technology Co., Beijing, China) is a colloidal gold lateral flow assay for the qualitative detection of IgM and IgG antibodies to undisclosed SARS-CoV-2 epitopes of N and S proteins. In accord with the manufacturer’s instructions, 10 μl of preserved serum was placed into the test kit, followed by the buffer. The result was available 15 min later. This immunochromatographic test is hereinafter referred to as the ‘IC’ test ^(6)^.

#### Electrochemiluminescence immunoassay (ECLIA)

An electrochemiluminescence immunoassay (ECLIA) using the Elecsys^Ⓡ^ Anti-SARS-CoV-2 assay and the cobas e801 module (Roche Diagnostics, Rotkreuz, Switzerland) is a double-antigen sandwich assay for the qualitative detection of different types of antibodies against SARS-CoV-2 nucleocapsid (N) proteins, primarily IgG^4^. This ECLIA was carried out according to the manufacturer’s instructions, and a cut-off index (COI) ≥1.0 indicates a positive diagnosis. The reagents for 5,000 samples were obtained in collaboration with Roche, and the reagents for the other samples were purchased from Roche.

#### Chemiluminescent enzyme immunoassay (CLEIA)

A chemiluminescent enzyme immunoassay (CLEIA) was performed by an HISCL-5000 immunoassay analyzer (Sysmex, Kobe, Japan) for the qualitative detection of IgG against SARS-CoV-2 nucleocapsid protein (N-IgG) and spike protein (S-IgG), respectively. This was carried out according to the manufacturer’s instructions, but during the survey, the cut-off was optimized and changed to stricter standards (from N-IgG 10 signal unit (SU) /ml and S-IgG 20 SU/ml to N-IgG 20 SU/ml and S-IgG 35 SU/ml). The result was considered positive if N-IgG or S-IgG was positive. This test is hereinafter referred to as the CLEIA, CLEIA-N, or CLEIA-S. The measurement of antibody titers was done by Sysmex under an outsourcing agreement.

#### Measurement of neutralizing activity against SARS-CoV-2

The neutralizing activity of each serum sample was evaluated by a neutralization test against SARS-CoV-2 (Biken-2 strain) in a biosafety level 3 laboratory, which was kindly provided by the Research Foundation for Microbial Diseases of Osaka University (BIKEN), Osaka University. At 24 hr before the assay, 4 × 10^4^ Vero E6 (TMPRSS2) cells per well were seeded in 96-well tissue culture microplates ^(7)^. A two-fold serial dilution of heat-inactivated (56°C, 30 min) serum was prepared using Dulbecco’s Modified Eagle’s Medium as the diluent and mixed with a 100 tissue culture infectious dose (TCID)_50_ of virus and incubated at 37°C for 1 hr. After this incubation, the serum-virus mixture was added to Vero E6 (TMPRSS2) cells and incubated at 37°C for 3 days. The neutralizing antibody titer was determined as the highest serum dilution that did not show any cytopathic effects by observation under a light microscope.

### Sample analysis

The obtained sera were first analyzed by the ECLIA and CLEIA. The sera that were positive by either of or both the ECLIA and CLEIA were then analyzed by the IC test and the neutralizing assay against SARS-CoV-2. The sera which were negative by both of the ECLIA and CLEIA were considered negative, and they were not subjected to the IC test or neutralizing assay.

This study was approved by the ethical committees of Kobe University Graduate School of Medicine (approval code: B2056703), and all institutions enrolled (Kobe University Hospital, anonymous X Hospital, Hyogo Prefectural Nishinomiya Hospital, Hyogo Prefectural Amagasaki General Medical Center, Hyogo Prefecture Health Promotion Association, and Hyogo Prefectural Kobe Children’s Hospital) were granted the approval. Regarding X Hospital and Hyogo Prefectural Health Promotion Association, ethical approval was done under the ethical committee of Kobe University Graduate School of Medicine. The other facilities also got approval from their internal ethical review committees. This study was a retrospective observation study, and carried out by the opt-out method of each institution’s website (Kobe University Hospital, anonymous X Hospital, Hyogo Prefectural Nishinomiya Hospital, Hyogo Prefectural Amagasaki General Medical Center, Hyogo Prefecture Health Promotion Association and Hyogo Prefectural Kobe Children’s Hospital).

## Results

A total of 10,377 serum samples of the in- and outpatients were analyzed for the seroprevalence of COVID-19. There were no duplicate samples from one individual. All of the samples were analyzed by the ECLIA, but the samples from Hyogo Prefectural Kobe Children’s Hospital were not analyzed by the CLEIA due to the small amount of serum.

The patients’ characteristics and the number of positive samples at each hospital are shown in Table 1: 5,184 males (49.9%) and 5,193 females (50.1%), with the median age of 62 years, including patients aged 0 to 99 years. Twenty-seven cases (0.26%) were positive for SARS-CoV-2 by the ECLIA, and 51 cases (0.49%) were positive for SARS-CoV-2 by the CLEIA. Seventeen cases were positive on both the CLEIA-N and -S, whereas 29 cases were positive on the CLEIA-N and 39 cases were positive on the CLEIA-S.

**Table 1.** Patients’ Characteristics and the Number of Positive Cases in Each Hospital.

	Total (N=10377)	Kobe university hospital (Ko) (N=2048)	anonymous X Hospital (X) (N=2000)	Hyogo Prefectural Nishinomiya Hospital (NI) (N=2010)	Hyogo Prefectural Amagasaki General Medical Center (AM) (N=2000)	Hyogo Prefecture Health Promotion Association (HE) (N=1000)	Hyogo prefectural Kobe Children’s Hospital (CH) (N=1319)
Male - no.(%)	5184 (49.9)	1045 (51)	989 (49.4)	904 (44.9)	1001 (50)	596 (59.6)	689 (52.2)
Median age - yr (range)	62 (0-99)	67 (2-96)	69 (1-95)	69 (0-99)	70 (1-97)	47 (19-82)	8 (0-19)
ECLIA positive - no.(%)	27 (0.26)	5 (0.24)	7 (0.35)	4 (0.19)	6 (0.3)	4 (0.4)	1 (0.07)
CLEIA positive (-N or -S) - no.(%)	51 (0.49)	12 (0.58)	12 (0.6)	9 (0.44)	11 (0.55)	7 (0.7)	ND
CLEIA-N positive - no.(%)	29 (0.27)	4 (0.19)	11 (0.55)	7 (0.34)	4 (0.2)	3 (0.3)	ND
CLEIA-S positive - no.(%)	39 (0.37)	12 (0.58)	6 (0.3)	7 (0.34)	10 (0.5)	4 (0.4)	ND
CLEIA-N and -S positive - no.(%)	17 (0.16)	4 (0.19)	5 (0.25)	5 (0.24)	3 (0.15)	0 (0)	ND
ECLIA and CLEIA positive - no.(%)	14 (0.13)	1 (0.05)	4 (0.2)	3 (0.15)	3 (0.15)	3 (0.3)	ND
ECLIA or CLEIA positive - no.(%)	64 (0.6)	16 (0.78)	15 (0.75)	10 (0.49)	14 (0.7)	8 (0.8)	1 (0.07)
IC positive - no.(%)	22 (0.21)	2 (0.09)	8 (0.4)	5 (0.24)	3 (0.15)	4 0.4)	0 (0)
Neutralization activity positive - no.(%)	16 (0.15)	2 (0.09)	4 (0.2)	3 (0.15)	3 (0.15)	4 (0.4)	0 (0)

ECLIA: electrochemiluminescence immunoassay, CLEIA: chemiluminescent enzyme immunoassay, CLEIA-N: CLEIA against severe acute respiratory syndrome coronavirus 2 (SARS-CoV-2) nucleocapsid protein, CLEIA-S: CLEIA against SARS-CoV-2 spike protein, IC: immunochromatographic test,

The 64 cases (0.6%) which were positive for SARS-CoV-2 by either of or both the ECLIA and CLEIA were analyzed further by the IC test and neutralization activity assay. The patients’ characteristics and the percentage of positive cases at each hospital were almost the same, with the exception of the Children’s Hospital (Table 1).

**Figure 2.**
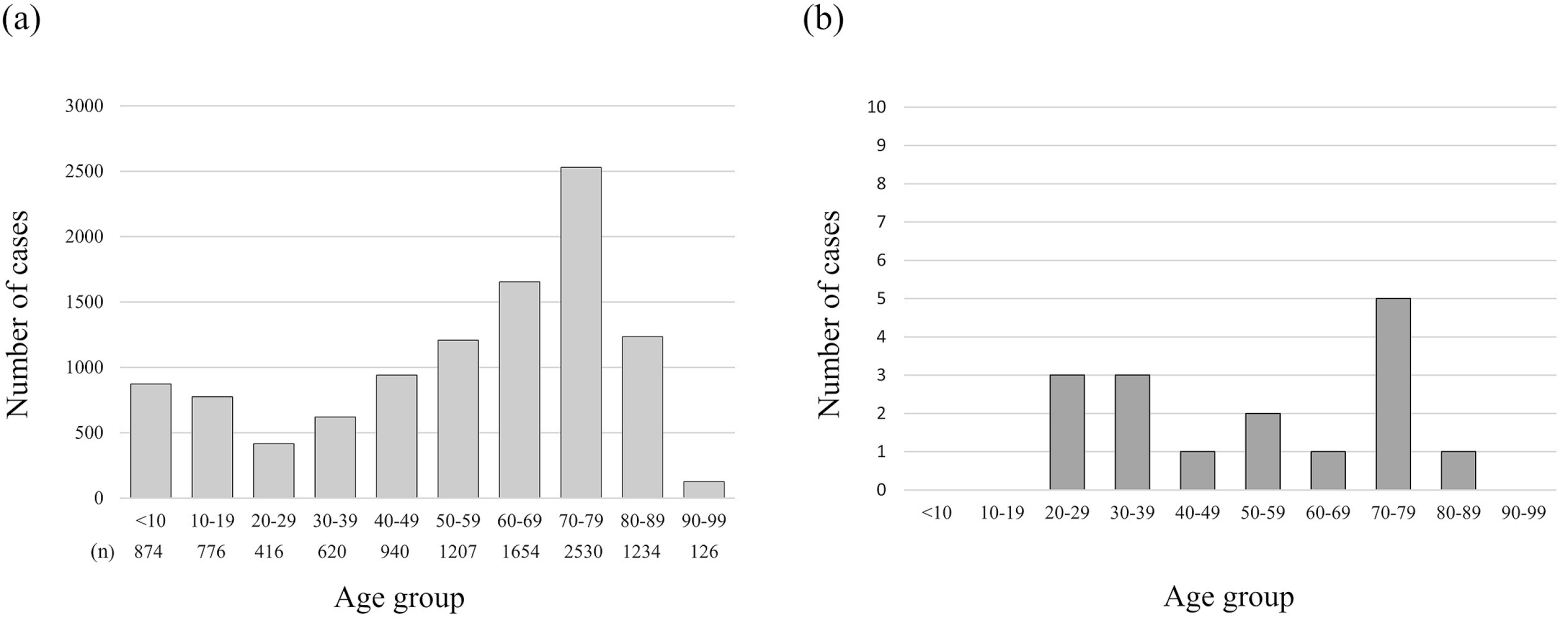
Case distribution of all patients and positive patients by age. (a) Distribution of all cases (n = 10,377) by age. (b) Distribution of the positive cases on neutralizing activity assay (n = 16) by age.

Figure 2 illustrates the distribution of all patients and positive cases by age. Although the number of patients was high in the 70s age group and low in the 20s and 30s groups, there were several positive cases in their 20s and 30s in addition to the 70s. Table 2 lists all positive patients by hospital. The results of the IC test and neutralizing activity assay for the 64 positive cases, which were classified based on the patterns of ECLIA, CLEIA, CLEIA-N and CLEIA-S results, are shown in Table 3. The IC test result was positive in 22 of the 64 patients (34%). Of note, nine triple-positive cases (positive for ECLIA, CLEIA-N and CLEIA-S) were also positive on the IC test. In the other patterns, the IC test’s positive rate tended to be low, whereas it was high (7 of 9 cases, 77%) in the CLEIA-N-positive group but ECLIA and CLEIA-S-negative. During this study, the CLEIA cut-off was optimized and changed to stricter criteria, and there were seven cases that were positive on the IC test among the patients whose result was changed from positive to negative due to this cut-off change (not included in Table 2).

**Table 2.**
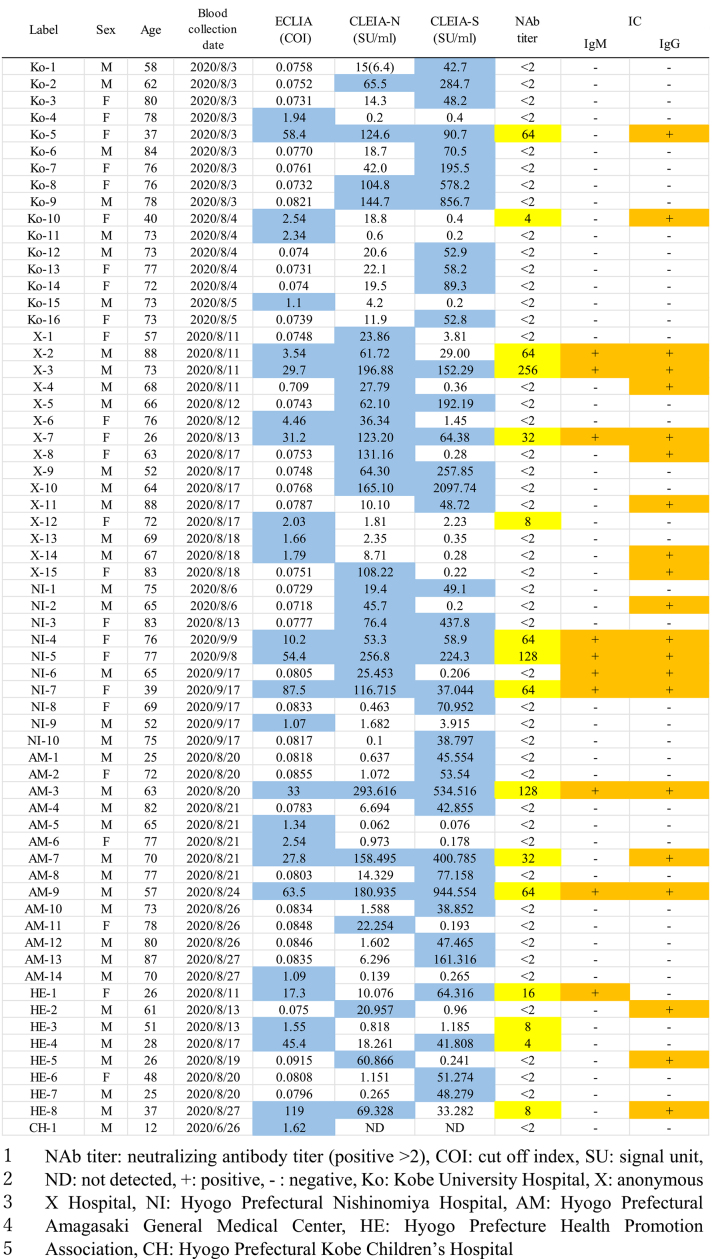
Results of Antibody Tests in 64 Individual Cases Which Were Positive on ECLIA 2 or CLEIA by Hospital.

**Table 3.** Results of Antibody Tests in 64 Cases Classified Based on the Patterns of ECLIA 2, CLEIA-N, and CLEIA-S.

	All (N=10377)	ECLIA or CLEIA positive (N=64)		Both positive (N=14)			Either positive (N=50)				
			ECLIA	+	+	+	－	+	－	－	+
			CLEIA-N	+	+	－	+	－	+	－	ND
			CLEIA-S	+	－	+	+	－	－	+	ND
				(N=9)	(N=3)	(N=2)	(N=8)	(N=12)	(N=9)	(N=20)	(N=1)
Male - no.(%)	5184 (49.9)	39 (60,9)		4 (44.4)	2 (66.6)	1 (50)	6 (75)	8 (66.6)	5 (55.5)	12 (60)	1 (100)
median age - yr (range)	62 (0-99)	70 (12-88)		63 (26-77)	76 (37-88)	27 (26-28)	70.5 (52-83)	69.5 (40-78)	65 (26-83)	74 (25-88)	12
median ECLIA (COI) (IQR)	0.0781 (0.0753-0.0818)	0.084 (0.076-2.39)		33 (29.7-58.4)	4.46 (4-61.7)	31.3 (24.3-38.3)	0.07 (0.07-0.07)	1.72 (1.28-2.1)	0.07(0.07-0.08)	0.07 (0.07-0.08)	1.62
median CLEIA N (SU/ml) (IQR)	0.1 (0.14-0.29)	19 (1.5-71)		158 (123-196)	61.7 (49-65.5)	14.1 (12.1-16.2)	76.4 (62.1-104)	1.32 (0.5-2.81)	27.7 (23.8-60.8)	1.36 (0.59-6.39)	ND
median CLEIA S (SU/ml) (IQR)	0.28 (0.14-0.63)	47 (0.68-83)		152 (64-400)	29 (15.2-31.1)	53 (47.4-58.6)	361 (241-647)	0.31 (0.2-0.59)	0.24 (0.2-0.36)	52 (46.9-70)	ND
IC positive - no.(%)	22 (0.21)	22 (34.3)		9 (100)	2 (66.6)	1 (50)	0 (0)	2 (16.6)	7 (77.7)	1 (5)	0 (0)
Neutralization activity positive - no.(%)	16 (0.15)	16 (25%)		9 (100)	2 (66.6)	2 (100)	0 (0)	3 (25)	0 (0)	0 (0)	0 (0)
median Neutralization antibody titer (IQR)	ND	< 2 (<2-3)		64 (64-128)	8 (<2 - 64)	10 (7-13)	< 2	< 2	< 2	< 2	< 2

Neutralization activity was observed in 16 of the 64 positive cases (25%), and these cases were not included in the ECLIA-negative group (Table 4). Fourteen cases were positive on both the ECLIA and CLEIA and showed high positivity rates on the IC test (12 of 14 cases, 85%) and neutralization activity assay (13 of 14 cases, 92.8%). Fifty cases were positive on either the ECLIA or the CLEIA and showed low positivity rates on the IC test (10 of 50 cases, 20%) and neutralization activity assay (three of 50 cases, 6%). There were three cases that were positive for neutralization activity but negative on the IC test. We calculated the positive rate of neutralizing antibody as the serum antibody prevalence; it was 0.15%.

**Table 4.** Results of the Neutralization Activity Titer and IC Test in 64 Individual Cases Classified Based on the Patterns of ECLIA, CLEIA-N and CLEIA-S.

	Both positive (N=14)									Either positive (N=50)														
ECLIA	+			+			+			－			+			－			－			+		
CLEIA-N	+			+			－			+			－			+			－			ND		
CLEIA-S	+			－			+			+			－			－			+			ND		
	(N=9)	NAb titer	IC	(N=3)	NAb titer	IC	(N=2)	NAb titer	IC	(N=8)	NAb titer	IC	(N=12)	NAb titer	IC	(N=9)	NAb titer	IC	(N=20)	NAb titer	IC	(N=1)	NAb titer	IC
	NI-4	64	+	X-2	64	+	HE-1	16	+	X-5	<2	－	NI-9	<2	－	AM-11	<2	－	AM-1	<2	－	CH-1	<2	－
	NI-5	128	+	X-6	<2	－	HE-4	4	－	X-9	<2	－	Ko-4	<2	－	HE-2	<2	+	AM-2	<2	－			
	NI-7	64	+	HE-8	8	+				X-10	<2	－	Ko-10	4	+	HE-5	<2	+	AM-4	<2	－			
	Ko-5	64	+							Ko-2	<2	－	Ko-11	<2	－	X-1	<2	－	AM-8	<2	－			
	X-3	256	+							Ko-8	<2	－	Ko-15	<2	－	X-4	<2	+	AM-10	<2	－			
	X-7	32	+							Ko-9	<2	－	X-12	8	－	X-8	<2	+	AM-12	<2	－			
	AM-3	128	+							NI-1	<2	－	X-13	<2	－	X-15	<2	+	AM-13	<2	－			
	AM-7	32	+							Ko-3	<2	－	X-14	8	－	X-2	<2	+	HE-6	<2	－			
	AM-9	64	+										HE-3	8	－	NI-6	<2	+	HE-7	<2	－			
													AM-5	<2	－				X-11	<2	+			
													AM-6	<2	－				Ko-1	<2	－			
													AM-14	<2	－				Ko-3	<2	－			
																			Ko-6	<2	－			
																			Ko-7	<2	－			
																			Ko-12	<2	－			
																			Ko-13	<2	－			
																			Ko-14	<2	－			
																			Ko-16	<2	－
																			NI-8	<2	－			
																			NI-10	<2	－			

NAb titer: neutralizing antibody titer (positive >2), IC: immunochromatographic test

## Discussion

Serological surveys are the best way to understand the spread of infectious diseases, especially in the presence of incomplete confirmations of asymptomatic cases ^(8)^. The clinical severity of COVID-19 varies from asymptomatic to severe, and it has been reported that approx. 40%-45% of individuals with COVID-19 are asymptomatic, and that such individuals can transmit SARS-CoV-2 to others for >14 days ^(9)^. Also, asymptomatic COVID-19 patients reportedly carry the same amount of virus as those who are symptomatic ^(10)^. Therefore, asymptomatic patients play a significant role in this pandemic, and several seroprevalence surveys have been conducted worldwide ^(11), (12), (13), (14), (15)^.

The findings from our present seroprevalence study for SARS-CoV-2 showed that the prevalence of antibodies is 0.15%. This result is not substantially different from the domestic survey in June 2020 revealing that the antibody prevalence was 0.03% to 0.17% in Miyagi, Tokyo and Osaka, indicating that most of the residents of those areas still did not have antibodies with neutralization activity against SARS-CoV-2 ^(5)^. That domestic survey was conducted using an ECLIA from Roche and a CLEIA that uses the ARCHITECT SARS-CoV-2 antibody detection kit (Abbott, Abbott Park, IL, USA), and revealed that neutralization activity was confirmed in all cases that were positive on both assay, and was not confirmed in cases that were positive on either assay ^(5)^. Our present investigation also revealed high positive rates on the IC test (85%, 12 of 14 cases) and neutralizing activity assay (13 of 14 cases) in the positive samples on both the ECLIA and the CLEIA. Thus, the diagnosis could become more specific by combining the ECLIA and the CLEIA. Since there was no neutralization activity in the ECLIA-negative group (Table 3), we speculate that the results obtained with the ECLIA may correlate more closely with neutralizing activity.

However, among the present 50 cases that were positive on either the ECLIA or the CLEIA, ten and three were positive on the IC test and neutralization activity assay, respectively. Unlike the previous report, this result did not confirm neutralization activity in cases that were positive on either the ECLIA or the CLEIA ^(5)^, suggesting a risk of overlooking positive results. Our results demonstrate the need for at least two methods of evaluating the antibodies against SARS-CoV-2. A neutralizing activity assay should confirm the multiple methods’ results. Even in facilities that cannot perform the neutralization activity test, patients with positive results on the two antibody tests might have neutralization activity, considering the high correlation between them shown in this study (Table 3 and 4).

In the 50 present cases (0.48%) that were positive on either the ECLIA or CLEIA, a false-positive result may mean two possibilities: a non-specific reaction or the possession of antibodies cross-reacted with SARS-CoV-2 antigens, e.g., antibodies for other coronaviruses but without neutralizing activity ^(16)^. Interestingly, we also detected several cases with high neutralizing activity, which may indicate a recent asymptomatic infection in the individuals, although there is the possibility of a past symptomatic infection. Since antibodies against SARS-CoV-2 gradually decrease ^(17)^, the positivity rate might be underestimated if the samples came from previously infected individuals who lost the antibodies.

The assessment of the neutralizing antibody is the most important evaluation for seroprevalence studies of antibodies because it is the most reliable method to ‘see’ the real infection. It is critical for the protection against virus infection. However, some of the people now infected with SARS-CoV-2 do not develop the neutralizing antibody for a long time. Thus some people have antibodies for the infection but do not have the neutralizing antibody ^(18)^. In the present study, nine cases (0.08%) were positive on the IC test but negative for neutralization activity. The antibodies without neutralizing activity for SARS-CoV-2 (especially for the spike protein) may cause antibody-dependent enhancement (ADE) and enhance the infection, resulting in the severe form of COVID-19.

On the other hand, three cases were positive for neutralizing antibodies but negative on the IC test. It is not easy to explain this result; however, some non-infected individuals reportedly carry neutralizing antibodies that react with SARS-CoV-2 spike protein ^(16)^. That report remains controversial. Cell-mediated immunity has also been important for virus protection ^(19)^. Thus, people who have the antibodies could be protected from the infection by cellular immunity even if they do not neutralize antibodies. Further investigations are required to test this concept.

Osaka, one of the endemic areas, is located in the east of Hyogo Prefecture. We conducted this study of patients at facilities from the west to the east of Hyogo Prefecture. There was no apparent difference in positivity rates among the six facilities, which might indicate that SARS transmission is stable at this stage and not expanding in Hyogo Prefecture. The stability might be due to social distancing. Since most Hyogo Prefecture residents do not infected with SARS-CoV-2, social distancing appears more effective in Hyogo than elsewhere in the world already affected by the pandemic.

Our study has several limitations. First, 1,319 patients aged 0 to 19 years at the Children’s Hospital could not be tested by the CLEIA, so our seroprevalence results for adolescents might thus be inaccurate. Second, we did not perform the IC test and the neutralization activity assay on patients who were negative on both the ECLIA and the CLEIA, but some of these patients may have been positive. Third, the antibody test’s blood collection period was not the same among the six facilities, at intervals of 2 to 6 weeks. This may have had some effects on the results.

The results revealed by our present analyses demonstrate that many people do not have antibodies for the SARS-CoV-2 infection, as they may be not infected or may no longer have the antibodies. Our findings contribute to understanding the seroepidemiology of COVID-19 in Japan today and could help predict the epidemics over the coming winter. The social measures that have been advised for the control of the infection, i.e., social distancing, mask-wearing in public, and thorough hand-washing, may also be reflected in our present data from Hyogo Prefecture.

## Conclusion

The SARS-CoV-2 antibody seroprevalence was 0.15% in this study, indicating that most of the people in the area investigated here still do not have the needed antibodies.

## Article Information

### Conflicts of Interest

Yasuko Mori received the reagents for 5,000 samples used here from Roche as a collaboration of this research.

### Sources of Funding

This work was partly supported by budget of Hyogo Prefectural Government.

### Acknowledgement

We thank Kazuro Sugimura MD, PhD (Executive Vice President, Kobe University) for his full support to promote this study. We express our sincere gratitude for cooperation and participation of staffs of Kobe University Hospital, Hyogo Prefectural Amagasaki General Medical Center, Hyogo Prefectural Nishinomiya Hospital, Hyogo Prefecture Health Promotion Association, anonymous X Hospital, and Hyogo Prefectural Kobe Children’s Hospital. We thank Dr. Tatsuya Nagano (Kobe University) for his assistance in preparation of document for the ethics. We thank Research Foundation for Microbial Diseases of Osaka University (BIKEN), Osaka University for providing SARS-CoV-2 strain.

### Author Contributions

All authors contributed to the concept of this article. KF drafted the manuscript; JA, MN and YM provided revisions; KF, JA, MN, LT and YM analyzed the data; KF, JA, MN, LT, AP, ZR, SA, JH, SS, YK, AN, SS, YT, KU, KM and IS did the experiments; YM and JS supervised the experiments; YI, KE, TO and YK collected the samples; YM conducted the project; All authors approved the final version of the manuscript.

Koichi Furukawa, Jun Arii, Mitsuhiro Nishimura and Lidya Handayani Tjan contributed equally to this work.

### Approval by Institutional Review Board (IRB)

This study was approved by the ethical committees of Kobe University Graduate School of Medicine (approval code: B2056703), and all institutions enrolled (Kobe University Hospital, anonymous X Hospital, Hyogo Prefectural Nishinomiya Hospital, Hyogo Prefectural Amagasaki General Medical Center, Hyogo Prefecture Health Promotion Association, and Hyogo Prefectural Kobe Children's Hospital) were granted the approval. Regarding X Hospital and Hyogo Prefectural Health Promotion Association, the ethics committee of Kobe University Graduate School of Medicine approved this study. The other facilities required and got approval from their internal ethical review committees. This was a retrospective observation study, and carried out by the opt-out method of each institution’s website (Kobe University Hospital, anonymous X Hospital, Hyogo Prefectural Nishinomiya Hospital, Hyogo Prefectural Amagasaki General Medical Center, Hyogo Prefecture Health Promotion Association, and Hyogo Prefectural Kobe Children's Hospital).

### Disclaimer

Yasuko Mori is one of the Editors of JMA Journal and on the journal's Editorial Staff. She was not involved in the editorial evaluation or decision to accept this article for publication at all.
